# Lost in translation? A multilingual Query Builder improves the quality of PubMed queries: a randomised controlled trial

**DOI:** 10.1186/s12911-017-0490-9

**Published:** 2017-07-03

**Authors:** Matthieu Schuers, Mher Joulakian, Gaetan Kerdelhué, Léa Segas, Julien Grosjean, Stéfan J. Darmoni, Nicolas Griffon

**Affiliations:** 1 Department of Biomedical Informatics, Rouen University Hospital, Rue de Germont, 76031 Rouen Cedex, France; 2 TIBS, LITIS EA 4108, Rouen University, Rouen, France; 3 Department of General Practice, Rouen University, Rouen, France; 4 INSERM, U1142, LIMICS, 75006 Paris, France

**Keywords:** Search engine, Medical information retrieval, PubMed/MEDLINE

## Abstract

**Background:**

MEDLINE is the most widely used medical bibliographic database in the world. Most of its citations are in English and this can be an obstacle for some researchers to access the information the database contains. We created a multilingual query builder to facilitate access to the PubMed subset using a language other than English. The aim of our study was to assess the impact of this multilingual query builder on the quality of PubMed queries for non-native English speaking physicians and medical researchers.

**Methods:**

A randomised controlled study was conducted among French speaking general practice residents. We designed a multi-lingual query builder to facilitate information retrieval, based on available MeSH translations and providing users with both an interface and a controlled vocabulary in their own language. Participating residents were randomly allocated either the French or the English version of the query builder. They were asked to translate 12 short medical questions into MeSH queries. The main outcome was the quality of the query. Two librarians blind to the arm independently evaluated each query, using a modified published classification that differentiated eight types of errors.

**Results:**

Twenty residents used the French version of the query builder and 22 used the English version. 492 queries were analysed. There were significantly more perfect queries in the French group vs. the English group (respectively 37.9% vs. 17.9%; *p* < 0.01). It took significantly more time for the members of the English group than the members of the French group to build each query, respectively 194 sec vs. 128 sec; *p* < 0.01.

**Conclusions:**

This multi-lingual query builder is an effective tool to improve the quality of PubMed queries in particular for researchers whose first language is not English.

## Background

Evidence based medicine is increasingly encouraged in medical practice and decision-making, which requires evidence based on valid research. MEDLINE, created by the US National Library of Medicine (NLM), is the most widely used medical bibliographic database in the world. It is the largest component of PubMed, which is the largest free online database of biomedical journal citations and abstracts. PubMed currently contains 26,415,890 citations from 5,650 indexed journals from 81 countries and in 60 languages. Each PubMed record is indexed with the NLM’s controlled vocabulary, the Medical Subject Headings (MeSH) [1].

More than 82% of PubMed citations are in English and this can be an obstacle for some researchers to access the information the database contains [2]. Nevertheless, some tools are, in fact, available to help non–native-English speakers to access PubMed references written in their native language: i.e. BabelMeSH [3, 4], Patient, Intervention, Comparison, Outcome (PICO) Linguist [4] and LiSSa [5]. Although some of these tools have demonstrated a high level of precision and coverage [5], they can only permit limited access to available evidence.

Recent research has also confirmed the lack of skills to perform a literature search among physicians: they not only are unable to master the specific querying process of medical databases but also feel uneasy in performing research [6]. The English used in the PubMed querying process might possibly explain some of the difficulties. Therefore, we were prompted to create a multilingual query builder to facilitate access to the PubMed subset using a language other than English (e.g. French, German, Spanish, or Norwegian), with an advanced multifunctional system. This practical tool relies on the MeSH translation in multiple languages to boost information retrieval.

The aim of this study was to assess the impact of a multilingual query builder on the quality of PubMed queries for physicians and medical researchers, in particular those whose first language is not English.

## Methods

### The multi-lingual Query Builder

**Fig. 1:**
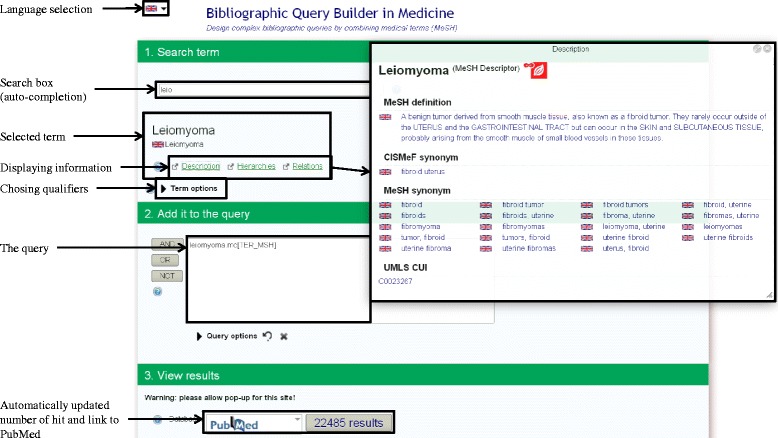
Query Builder web application detailed screenshot

Many users, including the French, have difficulties writing queries when performing research on PubMed. Therefore, we designed a multi-lingual query builder to facilitate information retrieval. Our objective was to develop an “easy-to-use” tool to select relevant MeSH terms. This tool is based on available MeSH translations, providing users with both an interface and a controlled vocabulary in their own language. Currently, the interface has been translated into French, English and Spanish while the MeSH thesaurus is available in more than fifteen languages (e.g. Dutch, English, Finnish, French, German, Italian, Portuguese and Spanish). Users can fill the search box with natural language. The Autocomplete search function helps to find the relevant MeSH terms. Full MeSH information is available, including definitions, relations and a hierarchical qualifiers list. The selected MeSH term is added to the query. Other MeSH terms can be added, using usual Boolean operators. When a satisfactory query is obtained, the user can submit it to PubMed. The tool allows to go back and forth: the user can observe the number of results given by the query - or the results itself - and reformulate the query if needed. An example for a given query appears in Fig. 1 (English interface). The query is then completed by using a wide range of synonyms such as natural language terms, in order to maximize recall and precision. As shown by Thirion et al., adding terms to the query significantly increases the precision when compared to the default query used by PubMed (54.5% vs 27%) [7].

The query builder is a web application (written in Java thanks to the Vaadin framework) connected to four services. Each service is dedicated to a specific task: i) the autocomplete function provides the MeSH terms related to the query; ii) the terminology server retrieval collects data of the selected MeSH term; iii) the InfoRoute service is the main application service, it builds the PubMed URL using the advanced PubMed search syntax (search tags [8], MeSH terms, Boolean operators) and iiii) a function to get the results number in PubMed for the generated query.

### Recruitment and study set-up

A randomised controlled study was conducted at the Department of General Practice of Rouen University during the month of January 2015. Native French speaking general practice residents were recruited by email by one researcher (MS) and randomly allocated either the French or the English version of the query builder web tool, using a computer-generated randomisation sequence. The residents were asked to translate 12 short medical questions (the same for each participant) into MeSH queries. The questions were written in French for both groups. They received a 15 minutes training about the query builder. During the training session, the participants used the query builder in the language for which they were randomized. This training was performed in French for all participants. It focused on the different stages of bibliographic search, the description and the use of MeSH thesaurus, Boolean operators and subheadings. The evaluation took place in two adjacent rooms of the Rouen University Medical School, at the same time. Residents allocated to the first room had access to the French version of the query builder, residents allocated to the second one had access to the English version.

Twelve short medical questions were designed with three levels of complexity. The clinical questions were drafted by an experienced medical librarian (GK). They were chosen arbitrarily in the logs of a document database developed in the Rouen University hospital (http://www.chu-rouen.fr/cismef/). They were then validated by two physicians (MS and NG) for medical relevance. The level of complexity was decided in regard to the number of MeSH terms (descriptors or subheadings) required for a correct query. Low difficulty questions required two terms. Medium difficulty questions required three terms. High difficulty questions required four terms. The difficulty to express an idea with MeSH terms was also taken into account for one question (salty taste in the mouth). The list of short medical questions is shown in Table 1.

**Table 1:** Short medical questions (EN/FR) and queries considered as correct

Low difficulty questions	
Fibroid uterus spontaneous rupture/Rupture spontané d’un fibrome utérin	Leiomyoma AND rupture, spontaneous
Alopecia areata prevention/Prévention des pelades	Alopecia areata/prevention and control
Vitamin D determination in blood/Dosage de la vitamine D dans le sang	Vitamin D/blood; vitamin D/analysis OR vitamin D/blood; vitamin D/blood OR (vitamin D AND blood chemical analysis)
Sarcopenia for over 65 years old patients/Sarcopénie chez les patients de plus de 65 ans	Sarcopenia AND aged
Medium difficulty questions	
Vaccination induced pain in infant/Douleur au cours de la vaccination des nourrissons	Infant AND Pain AND vaccination; infant AND (Pain OR pain measurement OR pain management) AND vaccination
Guidelines for breast cancer treatment/Recommandations sur le traitement du cancer du sein	Breast neoplasms/therapy AND practice guidelines as topic; breast neoplasms/therapy AND practice guideline
Asthma epidemiology in USA/Epidémiologie de l’asthme aux Etats-Unis	Asthma/epidemiology AND united states; (asthma/epidemiology OR (asthma AND epidemiology)) AND united states
Screening for uterine cervical neoplasm/Dépistage du cancer du col de l’utérus	Mass screening AND uterine cervical neoplasms/prevention and control; mass screening AND uterine cervical neoplasms/diagnosis;
High difficulty questions	
Salty taste in the mouth/Goût sale dans la bouche	Sodium chloride AND dysgeusia; sodium chloride AND taste disorders; sodium chloride AND taste perception
Allopurinol cutaneous side effect/Effets secondaires cutanés de l’allopurinol	Allopurinol/adverse effect AND skin diseases/chemically induced; allopurinol/adverse effect AND (skin diseases/chemically induced OR skin manifestations/chemically induced)
Glucocorticoids effects on asthmatic patient’s growth/Impact des glucocorticoïdes sur la croissance du patient asthmatique	((Glucocorticoids/adverse effects) AND (growth/drug effects OR growth disorders/chemically induced) AND asthma/drug therapy; ((glucocorticoids/adverse effects) AND (growth/drug effects OR growth disorders/etiology) AND asthma/drug therapy
Antibiotics dosage for overweight or obese patient/Posologie des antibiotiques chez le patient en surpoids ou obèse	Anti-bacterial agents/administration and dosage AND (Obesity OR overweight); (anti-bacterial agents/administration and dosage OR (drug dosage calculations AND anti-bacterial agents)) AND (Obesity OR overweight)

Questions were presented in French to all the participants. Queries only contain MeSH terms and/or MeSH terms/subheadings, except “practice guideline” which is a publication type

For evaluation purposes, the query builder was embedded in a light web application. It allowed investigators to lock the interface language (English vs. French), to propose one different page for each short medical question, record the submitted query and the overall response time. Participants were free to navigate between each medical question and change their queries.

### Outcomes and statistical analysis

The main outcome was the quality of the query. The expected queries were built by a team of medical librarians and are available in Table 1. Both groups delivered queries in English and these queries were then submitted to the librarians for evaluation. The query quality was assessed according to a modified published classification [9] that differentiated eight types of errors: irrelevant MeSH term, over-specification (descriptors or subheadings), under-specification (descriptors or subheadings), incorrect operator and syntax error. The description of these types of errors is shown in Table 2. Queries may present more than one mistake of each kind. If a query had two irrelevant MeSH terms and one under-specified qualifier, three errors were counted. Queries without any errors were considered as perfect. Two librarians blinded to the arm independently evaluated each query and resolved discrepancies by consensus. Proportions were compared using Fisher’s test. Analyses were stratified according to the difficulty level.

**Table 2:** Summary of main results

Error type and its description (from Vanopstal et al)	French version n (%)	English version n (%)	*p*
Perfect queries	89 (37.9)	46 (17.9)	<0.01
Irrelevant MeSH term	52 (22.1)	83 (32.3)	<0.01
Query contains at least one incorrect MeSH term			
Over-specification (descriptors)	28 (11.9)	39 (15.2)	0.3
Query contains at least one MeSH term that is too narrow			
Over-specification (qualifiers)	17 (7.2)	17 (6.6)	0.8
Under-specification (descriptors)	41 (17.4)	87 (33.9)	<0.01
Query contains at least one MeSH term that is too broad			
Under-specification (qualifiers)	48 (20.4)	72 (28)	0.05
Incorrect operator	1 (0.4)	1 (0.4)	1
Misuse of “AND” or “OR”			
Syntax error	2 (0.9)	4 (1.6)	0.7
Query contains unmatched brackets or quotes, or truncated words			

The time spent in building queries was measured as a secondary outcome. It was measured by the web form from the reading of the clinical question to the submission of the final MeSH query by the end-user. Times were compared using the Mann-Whitney test.

For an expected improvement of 15% in the group ‘native language’ vs. ‘English language’, from 25 to 40%, 17 end-users per group were required (alpha = 0.05, beta = 0.1, var = pq/n). All statistical tests were performed with R 3.0.2 software.

## Results

**Fig. 2:**
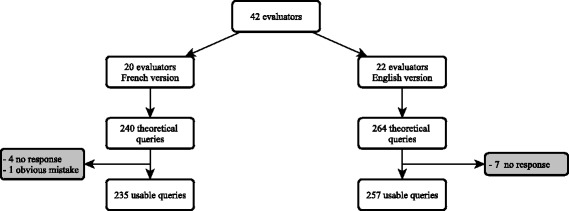
Application screenshot

Forty-two residents participated in this study, each of them had at least one year of experience. Twenty residents used the French version of the query builder and 22 used the English version. The mean age was 26.9 years old in the French group and 26.7 years old in the English group (*p* = 0.58). The proportion of male was 35% in the French group and 45.5% in the English group (*p* = 0.54). After the exclusion of 11 non-responses and one obvious mistake (query related to a previous question), 492 queries were analysed. Please see flow chart in Fig. 2.

Queries based on low difficulty questions contained 0.83 error on average [0.71–0.96], queries based on medium difficulty questions 0.92 error [0.80–1.03] and queries based on high difficulty questions 1.69 errors [1.53-1.85] (*p* < 0.01).

One hundred and thirty-five queries (27.4%) were considered as perfect. There were significantly more perfect queries in the French group vs. the English group (respectively 37.9% vs. 17.9%; *p* < 0.01). The most frequent errors were the use of an underspecified MeSH term (44.7%) or an irrelevant MeSH term (27.4%). Members of the French group performed significantly better for these two kinds of mistake, respectively 35.7% vs 52.9% (*p* < 0.01) and 22.1% vs. 32.3% (*p* = 0.01). No differences were found between the two groups concerning over-specification errors, the use of incorrect operator or syntax errors. See Table 2 for detailed results.

There were significantly more perfect queries in the French group vs. the English group for low difficulty questions (48.1% vs 31.8%; *p* = 0.04), medium difficulty questions (45.6% vs 18.4%; *p* < 0.01) and high difficulty questions (20.3% vs 3.5%; *p* < 0.01).

**Fig. 3:**
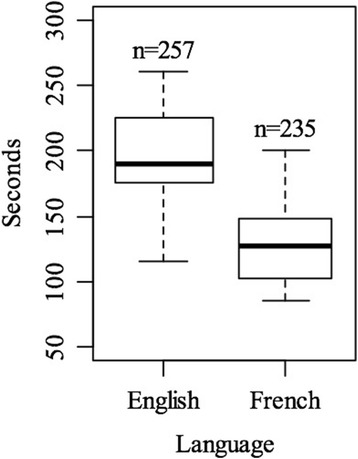
Distribution of average time taken by participants to write one query, according to language

The fastest evaluator spent 85 seconds on average for each query, the slowest 260 seconds. It took significantly more time for the English participants than the French participants to build each query, respectively 194 sec vs. 128 sec; p < 0.01 (Fig. 3).

## Discussion

### Summary of main results

Our research findings show that a multi-lingual query builder to access PubMed could be a useful tool in research and clinical practice for non-native English speakers. Participants querying in their first language built twice more perfect queries than participants querying in English. These results were found for low, medium and high difficulty questions. The impact of querying in first language increased with the level of difficulty. Participants querying in their first language took less time to build each query than participants querying in English.

### Discussion of the main results

Many barriers to query building and information retrieval among healthcare professionals have previously been identified in the literature. Currently, the most reported obstacles are: (i) the amount of time required to find information, (ii) difficulties in reformulating the original question and finding an optimal search strategy, (iii) lack of a good source of information, uncertainty as to whether all relevant information has been found and (iiii) inadequate synthesis of any pieces of evidence into a clinically useful approach [10, 11]. The literature also shows that physicians, and especially primary care doctors, express a need for database training, regardless of their first language [6, 12]. Physicians’ difficulties in building search queries are well known. In 2007, a web log analysis was undertaken in a meta-search engine covering 150 health resources and a variety of guidelines. It showed that most queries were built using a single search term and no Boolean operator [13]. A similar study was conducted on PubMed queries. If PubMed queries had a median of three terms, only 11% of them contained Boolean operators [14]. Many factors can influence the physicians’ ability to build relevant search queries, including the level of English skills [9, 15]. These findings suggest that our query builder may be of significant value for non-native English speaking healthcare professionals.

As previously mentioned, our data suggest that the impact of using the first language increases with the complexity of clinical questions. Complexity appears to play a key role in physicians’ difficulties in information retrieval. They fail to master the use of Boolean operators and, when dealing with complex clinical questions, GP trainees tend to refer to their colleagues more than electronic sources [13, 16].

Participants querying in their first language took less time to build each query than participants querying in English. This appears to be an important finding, as time constraints are always cited as a major obstacle when seeking information and may improve the PubMed’s use as searches with PubMed are not as frequent as searches with Google or UpToDate [10, 17].

Nevertheless, only one-third of the queries were considered perfect, even among participants querying in their first language. Irrelevant MeSH terms and the lack of specification in descriptors and subheadings lead to poor precision and recall [9]. Querying in first language will not solve all the problems faced by researchers and physicians, especially as the overwhelming majority of PubMed references remain in English. Some research tools already provide an automatic translation of a biomedical text, including titles and abstracts and using the MEDLINE database [18]. Other tools increase the information retrieval task performance by allowing non–native-English speakers to access PubMed references written in their native language: BabelMeSH [3, 4], Patient, Intervention, Comparison, Outcome (PICO) Linguist [4] and LiSSa [5]. According to Gagnon et al., educational meetings currently seem to be the only type of interventions showing a significant positive effect on clinical information retrieval technologies adoption by healthcare professionals [19].

### Strengths and limitations

This study has several limitations. First, only the quality of the query was assessed and not the quality of the results. However, the quality of the query is strongly associated to the quality of the results. Vanopstal et al. demonstrated that under-specified queries led to an increase of noise and our data show that under-specification is the main error compensated by querying in users’ first language [9]. An evaluation of the queries is planned as a second step. A sample of discrepant results (using queries built by English group versus French group) will be rated by a group of physicians. This will allow us to assess the impact of the multilingual query builder on the quality of the results. Second, this study only involves French residents in general medicine and this could affect the external validity of the results. Nevertheless, we do know that PubMed querying issues are encountered among physicians and medical researchers worldwide [14]. In order to enhance external validity, a similar trial will soon be conducted among Spanish speaking residents and physicians at the Buenos Aires Italian Hospital (Argentina).

This study is, to our knowledge, the first published evaluation of a multi-lingual query builder to access the PubMed subset. In order to avoid any before and after studies bias, a randomised controlled trial was carried-out. The clinical questions were drafted by an experienced medical librarian (GK) and validated by two physicians (MS and NG). The theoretical difficulty levels of the clinical questions were proven due to the significant association between the average number of errors and the difficulty level of the questions. The assessment of the queries was made independently by two librarians (GK and LS), using a modified published classification [9].

### Perspectives

As might be expected, this study clearly demonstrates that querying in first language is easier than querying in English. This study will soon be repeated among Argentinean healthcare professionals, comparing the use of Spanish and English. The multi-lingual query builder permits to overcome the obstacle of English when building queries, and could be of major interest for students, clinicians and researchers worldwide.

The query builder is already available in more than fifteen languages including Dutch, English, Finnish, French, German, Italian, Portuguese and Spanish. Some translations of MeSH terms or of the web site interface may still be lacking and we actively encourage all the teams working on MeSH translation, i.e. INSERM and Inist-CNRS in France. Our tool permits physicians and medical researchers to perform a request in the most relevant PubMed database fields. Full MeSH information is available, including definitions, relations and a hierarchical qualifiers list. A wide range of synonyms is also used automatically as natural language terms to complete the query, in order to maximize recall. Some features are currently lacking, like combining previous requests, however our priority was to first build an effective easy-to-use tool.

## Conclusion

Physicians often feel incompetent when seeking medical information, especially when using bibliographic databases. This sentiment is sometimes associated with a feeling of illegitimacy, as these databases were not created to meet their needs. This shows that there is a gap between an idealized academic search model and the practical requirements of everyday life [20]. This multi-lingual query builder is an effective tool to improve the quality of PubMed queries and should narrow the gap, particularly for physicians and researchers whose first language is not English.

## References

[1] Lipscomb CE (2000). Medical Subject Headings (MeSH). Bull Med Libr Assoc.

[2] Sheets L, Gavino A, Callaghan F, Fontelo P (2013). Do language fluency and other socioeconomic factors influence the use of PubMed and MedlinePlus?. Appl Clin Inform.

[3] Liu F, Ackerman M, Fontelo P. BabelMeSH: development of a cross-language tool for MEDLINE/PubMed. AMIA Annu Symp Proc AMIA Symp. 2006;1012 PMC1839504 17238631

[4] Fontelo P, Liu F, Leon S, Anne A, Ackerman M (2007). PICO Linguist and BabelMeSH: development and partial evaluation of evidence-based multilanguage search tools for MEDLINE/PubMed. Stud Health Technol Inform.

[5] Griffon N, Schuers M, Soualmia LF, Grosjean J, Kerdelhué G, Kergourlay I (2014). A search engine to access PubMed monolingual subsets: proof of concept and evaluation in French. J Med Internet Res.

[6] Schuers M, Griffon N, Kerdelhue G, Foubert Q, Mercier A, Darmoni SJ (2016). Behavior and attitudes of residents and general practitioners in searching for health information: From intention to practice. Int J Med Inf.

[7] Thirion B, Robu I, Darmoni SJ (2009). Optimization of the PubMed automatic term mapping. Stud Health Technol Inform.

[8] Cimino JJ, Elhanan G, Zeng Q (1997). Supporting infobuttons with terminological knowledge. JAMIA.

[9] Vanopstal K, Buysschaert J, Laureys G, Vander Stichele R (2013). Lost in PubMed. Factors influencing the success of medical information retrieval. Expert Syst Appl.

[10] Coumou HCH, Meijman FJ (2006). How do primary care physicians seek answers to clinical questions? A literature review. J Med Libr Assoc.

[11] Davies K, Harrison J (2007). The information-seeking behaviour of doctors: a review of the evidence. Health Inf Libr J.

[12] Bryant SL (2004). The information needs and information seeking behaviour of family doctors. Health Inf Libr J.

[13] Meats E, Brassey J, Heneghan C, Glasziou P (2007). Using the Turning Research Into Practice (TRIP) database: how do clinicians really search?. J Med Libr Assoc.

[14] Herskovic JR, Tanaka LY, Hersh W, Bernstam EV (2007). A day in the life of PubMed: analysis of a typical day’s query log. J Am Med Inform Assoc.

[15] Vanopstal K, Stichele RV, Laureys G, Buysschaert J (2012). PubMed searches by Dutch-speaking nursing students: The impact of language and system experience. J Am Soc Inf Sci Technol.

[16] Magin P, Morgan S, Wearne S, Tapley A, Henderson K, Oldmeadow C (2015). GP trainees’ in-consultation information-seeking: associations with human, paper and electronic sources. Fam Pract.

[17] Thiele RH, Poiro NC, Scalzo DC, Nemergut EC (2010). Speed, accuracy, and confidence in Google, Ovid, PubMed, and UpToDate: results of a randomised trial. Postgrad Med J.

[18] Yepes AJ, Prieur-Gaston E, Névéol A (2013). Combining MEDLINE and publisher data to create parallel corpora for the automatic translation of biomedical text. BMC Bioinformatics.

[19] Gagnon M-P, Pluye P, Desmartis M, Car J, Pagliari C, Labrecque M (2010). A systematic review of interventions promoting clinical information retrieval technology (CIRT) adoption by healthcare professionals. Int J Med Inf.

[20] Younger P (2010). Internet-based information-seeking behaviour amongst doctors and nurses: a short review of the literature. Health Inf Libr J.

